# Iatrogenic Splenic Laceration Presenting as Syncope

**DOI:** 10.1155/2018/7639527

**Published:** 2018-06-03

**Authors:** Dhanalakshmi Thiyagarajan, Rebecca Jeanmonod

**Affiliations:** Department of Emergency Medicine, St. Luke's University Health Network, Bethlehem, PA, USA

## Abstract

Millions of colonoscopies are performed annually for routine health maintenance in the United States. Patients commonly have adverse events from colonoscopy preparation, anesthesia, and procedural complications. We report a case of syncope secondary to iatrogenic splenic laceration from colonoscopy.

## 1. Case

A 70-year-old man presented to the emergency department (ED) after falling twice at home. While standing after eating a light meal, he had two separate episodes of brief loss of consciousness. On the second fall, the patient had hit his right shoulder, cheek, and foot, prompting the visit. The patient had received a colonoscopy earlier in the day for routine cancer screening. He had followed proper protocol regarding his bowel prep and had not had any immediate complications related to the procedure or anesthesia. Since the colonoscopy, he had continuous bouts of cramping abdominal pain. He had also felt dizzy throughout this period but attributed it to dehydration related to his bowel prep. The patient denied striking his head, chest pain, shortness of breath, nausea, vomiting, or blood in his stools. His past medical history was significant for coronary artery disease, hyperlipidemia, and hypertension. The patient took his benazepril, aspirin, and atorvastatin on the day prior to colonoscopy.

On physical exam, the patient was afebrile with a heart rate of 87 and blood pressure of 130/78. The patient's head, neck, chest, and neurological exams were entirely normal. His abdominal exam was remarkable for tenderness in the right and left lower quadrant with some voluntary guarding, but no masses or rebound was appreciated. His orthopedic exam demonstrated tenderness to his right acromioclavicular joint and ecchymosis of his right 5th metatarsal with no deformity and normal range of motion at all joints.

**Figure 1:**
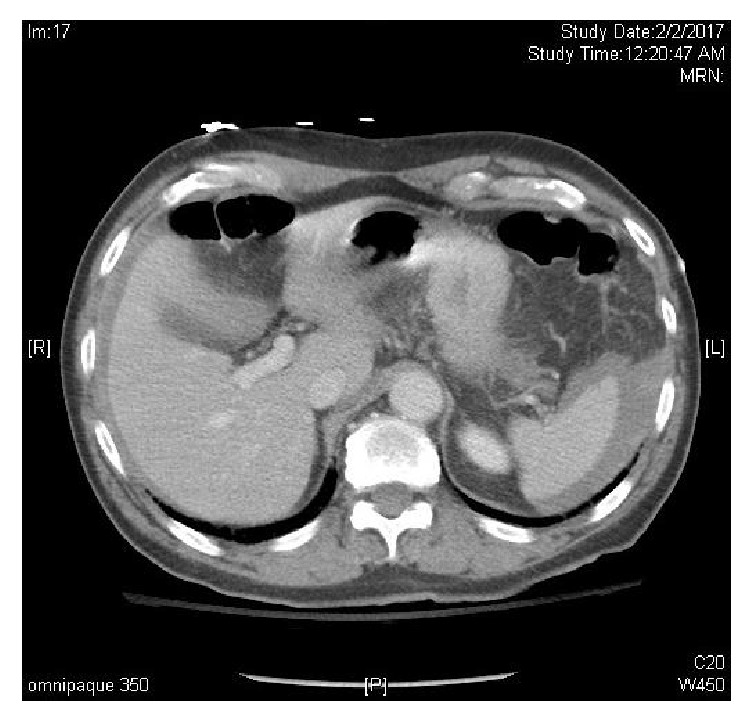
Horizontal CT scan demonstrating hemoperitoneum from iatrogenic spleen injury.

**Figure 2:**
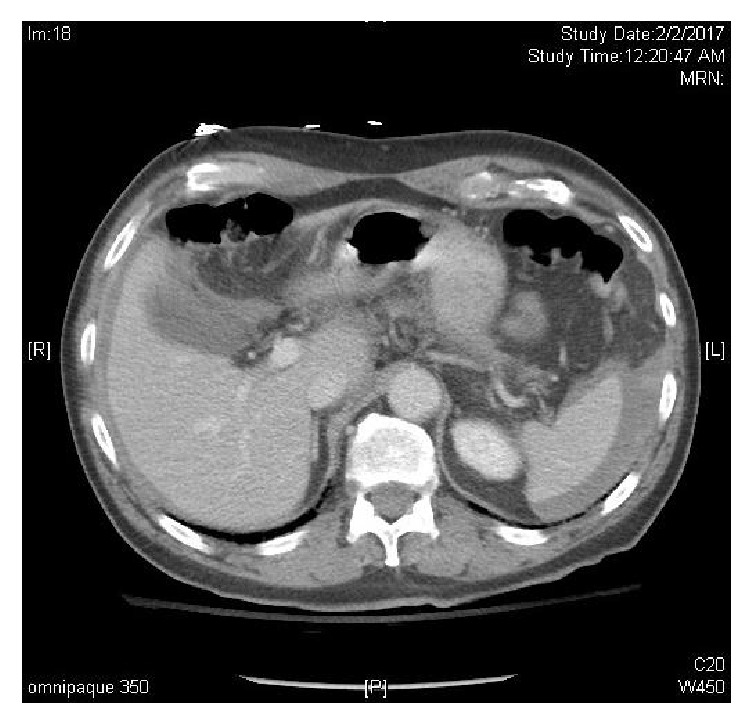
Horizontal CT scan demonstrating hemoperitoneum from iatrogenic spleen injury.

**Figure 3:**
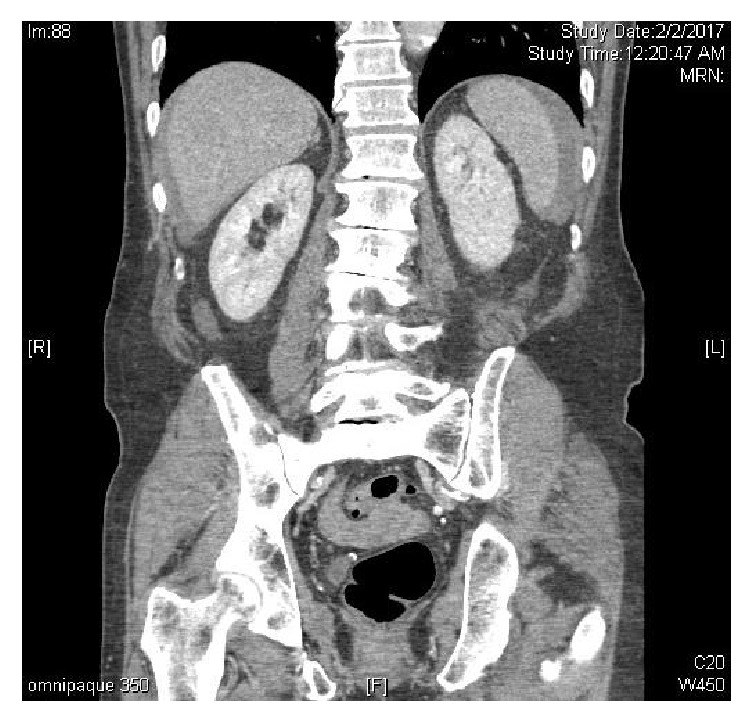
Coronal CT scan demonstrating hemoperitoneum from iatrogenic spleen injury.

On laboratory evaluation, the patient's hemoglobin was 12.4 g/dL, with normal platelets and chemistry studies. Head computed tomography (CT) and shoulder and chest radiography were normal. Foot radiography demonstrated a nondisplaced 5th metatarsal fracture. CT of the abdomen and pelvis demonstrated moderate hemoperitoneum with splenic laceration (Figures 1, 2, and 3).

The patient was admitted to the intensive care unit and underwent serial abdominal exams and every four-hour hemoglobin measurements. His hemoglobin fell to 8 g/dL at 48 hours after admission but subsequently stabilized. The patient was observed in the hospital and ultimately discharged home on hospital day 3 in good condition.

## 2. Discussion

Colonoscopies are recommended by the Centers for Disease Control (CDC) for routine screening for colorectal cancer in individuals over 50 years of age. Colonoscopy is also the test of choice to determine the source of lower gastrointestinal (GI) bleeding and is frequently performed to evaluate patients with abdominal pain or change in bowel habits. As such, in 2012, an estimated 15 million colonoscopies were performed in the United States [1]. As efforts to improve screening protocols continue and as the population continues to age, the number of colonoscopies performed annually is expected to rise.

Although not considered a high-risk procedure, colonoscopy commonly has complications related to bowel preparation, anesthesia, and the procedure itself. The typical bowel preparation regimen includes drinking a large volume of diarrhetic solution such as polyethylene glycol or hyperosmotic solution such as sodium phosphate. Bowel prep is generally well tolerated by young and healthy patients but can cause caloric deficits, dehydration, and electrolyte imbalance [2–5]. Additionally nausea, vomiting, and abdominal pain are reported in more than half of patients [3, 4]. Sodium phosphate preparations are lower in volume (improving tolerability for most patients) but should be used with caution in patients with renal disease or processes affecting electrolytes, as these osmotic agents can cause life-threatening electrolyte abnormalities [4, 5].

Complications related to anesthetic use are typically transient but may occur in over 1/3 of individuals and vary depending on agent used [6, 7]. Common complications include apnea, bradycardia, and hypotension [6, 7]. A full discussion of anesthetic complications is beyond the scope of this article.

The most common complication from the colonoscopy procedure itself is transient bloating and abdominal pain, occurring in 33% of patients [8]. These symptoms tend to be minor, benign, and self-resolving [8]. Serious complications, including respiratory arrest, cardiac arrhythmias, myocardial infarction, shock, colonic perforation, infection, hemorrhage, postpolypectomy syndrome, and death, arise in 2.8 per 1,000 procedures [8, 9]. Rarely, appendicitis, diverticulitis, subcutaneous emphysema, and tearing of mesenteric vessels may occur [9].

Splenic injury is a very rare complication of colonoscopies, with an approximate incidence of 1 in 100,000 since the first reported case in 1974 [9–11]. It is probable that splenic injury is an underreported complication of colonoscopy, as many patients have abdominal discomfort and may not undergo thorough evaluation for etiology [11]. Since splenic injuries may be stable and managed conservatively, this subset of patients may have resolution of their symptoms without detection and subsequent reporting of splenic injury. The most common splenic injury encountered is subcapsular hematoma [11]. Splenic laceration with hemoperitoneum, as occurred in our patient, is less common.

Splenic injury after colonoscopy is more common in female patients, accounting for about 74% of reported cases [11]. It has been reported in people aged 29–90, with an average age of 62 [11]. Although no clear relationship has been demonstrated between splenic injury and indication for colonoscopy, there is a correlation between injury and technical difficulty during the colonoscopy itself. Specific technical limitations linked to iatrogenic splenic injury include difficulty passing the colonoscope, looping of the instrument, traction on the splenocolic ligament, adhesions between the colon and the spleen, and presence of a large polyp or mass at the splenic flexure [9]. Techniques that appear to increase the risk of iatrogenic splenic injury include excess external pressure on the left hypochondrium to assist in colonoscope passage, which can cause blunt trauma to the area; hooking the splenic flexure to straighten the left colon or other maneuvers that increase traction at the splenic flexure; and keeping the patient in a supine rather than left lateral decubitus position [9]. Although there is no demonstrated risk to patients with prior trauma or intraabdominal surgery, women with prior pelvic surgery appear to be at greater risk, as do patients with history of smoking, chronic pancreatitis, and inflammatory bowel disease [11, 12].

Signs and symptoms of splenic injury can be vague and nonspecific. Most patients present within 24 hours of colonoscopy, although there are cases reported of later presentations [9, 11]. Therefore, a high index of suspicion is warranted in patients with recent colonoscopy. The most common complaint is nonspecific abdominal pain [9]. Patients may have left upper quadrant pain with referred left shoulder pain (Kehr's sign), but this is an insensitive finding [12]. Signs and symptoms related to hemorrhage, such as hypotension or syncope, as in our patient, may lead the provider to evaluate the patient for cardiac or infectious etiologies of his/her symptoms. Since hemoglobin levels may not fall for 6–24 hours after splenic injury, the provider should not rely upon normal hemoglobin to rule out splenic injury [12]. Other laboratory findings such as leukocytosis are nonspecific and unhelpful. In the stable patient, the gold standard for diagnosing a splenic injury is a contrast-enhanced CT scan. Diagnosis in the unstable patient may be made intraoperatively or in the angiography suite [11].

Management of these patients is tailored to their clinical presentations. All patients with known or suspected splenic injury should undergo resuscitation, with establishment of large bore intravenous access and blood products on hold to be used as needed. They should be admitted to a hospital capable of emergency operative intervention if their status worsens. Patients who are hemodynamically unstable require immediate intervention with either operative splenectomy or angiographic embolization of their splenic arteries. Stable patients may be observed as inpatients with serial abdominal exams and hemoglobin measurements to detect progression of their splenic injury. Many of these patients will remain stable and have no further sequelae, as occurred with our patient.

Splenic injury is an uncommon but dangerous complication of colonoscopy. The provider should be aware of this potential complication and have a low threshold for diagnostic imaging in patients with nonspecific abdominal complaints or symptoms of hypovolemia after colonoscopy.
